# A neurotoxic regimen of methamphetamine exacerbates the febrile and neuroinflammatory response to a subsequent peripheral immune stimulus

**DOI:** 10.1186/1742-2094-7-82

**Published:** 2010-11-22

**Authors:** Jessica B Buchanan, Nathan L Sparkman, Rodney W Johnson

**Affiliations:** 1 Laboratory of Integrative Immunology and Behavior, Department of Animal Sciences, University of Illinois Urbana-Champaign, 1207 W. Gregory Drive, Urbana, IL 61801, USA

## Abstract

Methamphetamine (MA) use is associated with activation of microglia and, at high doses, can induce neurotoxicity. Given the changes in the neuroinflammatory environment associated with MA, we investigated whether MA administration would interfere with the thermoregulatory and neuroinflammatory response to a subsequent peripheral immune stimulus. C57BL6/J mice were given four i.p. injections of either 5 mg/kg MA or saline at two hour intervals. Twenty-four hours following the first MA injection, mice were given 100 μg/kg LPS or saline i.p. and blood and brains were collected. Here we report that mice exposed to MA developed higher fevers in response to LPS than did those given LPS alone. MA also exacerbated the LPS-induced increase in central cytokine mRNA. MA alone increased microglial Iba1 expression and expression was further increased when mice were exposed to both MA and LPS, suggesting that MA not only activated microglia but also influenced their response to a peripheral immune stimulus. Taken together, these data show that MA administration exacerbates the normal central immune response, most likely by altering microglia.

## Background

Methamphetamine is a popular drug of abuse that has several central effects and is associated with an increased prevalence of HIV infection, hepatitis B and C, fungal infections, and possibly others [1-4]. Given the popularity of MA, it is becoming increasingly important to understand the effects of MA use on host responses. Among some of its detrimental effects, MA exposure has been associated with oxidative stress and the production of reactive oxygen and nitrogen species [5,6], neurotoxicity [7,8], and an increase in microglial activation and astrocytes, leading to the production of proinflammatory cytokines [9,10]. Long-term MA users show prominent microglial activation in certain brain regions that are sometimes apparent almost 2 years after MA use had ceased [9]. MA-induced microglial activation has also been shown in animals given high doses of MA [11-13] and there is evidence that this activation remains apparent up to seven days after treatment [10]. Both high and low doses of MA can induce inflammatory cytokines such as tumor necrosis factor α (TNFα), interleukin-1β (IL-1β), and IL-6 in the brains of rodents [14-17]. Indeed, high-dose MA administration increased TNFα protein in the straitum three days after treatment [18] and in the hippocampus seven days after treatment [10].

Peripherally, acute and chronic MA administration have been shown to reduce the number of leukocytes as well as NK cell activity of splenic lymphocytes [19]. Chronic MA administration reduced Con A-induced T-cell proliferation as well as IL-2 and IFNγ production in mouse splenocytes [20], and a binge dosing regimen suppressed the peripheral immune response to fungal infection [21]. MA has also been shown to decrease IL-1 production by splenocytes in mice [22] and reduce IFNγ and IL-10 while increasing IL-4, MCP-1, and TNFα in plasma [12]. Taken together, these data suggest that MA alters the production of both inflammatory and anti-inflammatory cytokines in the periphery while inducing a heightened inflammatory environment in the brain. This MA-induced increase in brain inflammation has the potential to influence the body's response to a subsequent immune stimulus.

While there are many studies concerning the consequences of MA use alone, little is known concerning the neuroinflammatory consequences of MA use and subsequent peripheral immune stimulation. Infection in the periphery results in the production of inflammatory cytokines which, through neural and humoral pathways, induce glial cells in discrete brain regions to produce the same inflammatory cytokines [23,24]. It is this central production of inflammatory cytokines that is responsible for inducing sickness behavior including fever, anorexia, as well as reduced locomotor and social behaviors. Excessive production of inflammatory cytokines has been shown to produce severe behavioral deficits and promote neurotoxicity [25]. Thus, circumstances that enhance inflammatory cytokine production by microglial cells, such as MA use, are likely to lead to pronounced and prolonged behavioral deficits that are not conducive to health and recovery.

We have previously shown that repeated administration of low-dose MA attenuated both the febrile and neuroinflammatory response to a subsequent injection of lipopolysaccharide (LPS) in mice [26]. The attenuated response appeared to be related to a MA-associated alteration in the microglial response. Mice exposed to five days of consecutive low-dose MA administration showed decreased microglial activation when given a subsequent injection of LPS compared to mice given LPS alone. Given this decreased microglial activity, we concluded that the MA-induced attenuation of the febrile and neuroinflammatory response was due to an inability of microglia to respond to LPS. Because of this, the present study utilized a MA administration schedule expected to activate microglia [7,13]. We hypothesized that this MA schedule would activate microglia and exacerbate the sickness and neuroinflammatory response to LPS. Here we report that exposure to MA enhances the LPS-induced fever response that is paralleled by increased microglial staining and a dramatic increase in inflammatory cytokine mRNA expression in the brain.

## Methods

### Animals and surgery

Adult (3-5 mo) male C57BL/6 mice from The Jackson Laboratories were used. Mice were housed in polypropylene cages and maintained at 23°C under a diurnal 12 h light-dark cycle (lights on at 0700) with *ad libitum *access to water and rodent chow. To allow for continuous monitoring of body temperature (Tb) and locomotor activity, mice were anesthetized with an intraperitoneal (i.p.) injection of ketamine and xylazine (100 and 10 mg, respectively) and implanted with a biotelemetry device (E-mitter, Mini Mitter, Bend, OR). Briefly, a midline abdominal incision was made 1 cm below the diaphragm and the E-mitter was positioned in the abdominal cavity along the sagittal plane. The muscle wall was sutured with chromic gut and the skin sutured with silk surgical thread. Following surgery, mice were individually housed and each cage was placed on a receiver board (model ER-4000 Receiver, Mini Mitter). Data was collected every 5 min utilizing the Vital View data acquisition system (Mini Mitter Co., Bend, OR). Tb and locomotor activity were monitored from the time of implantation until the end of the experiment. Mice were allowed at least 7 days to recover from surgery before any experimental procedures took place. All procedures were in accordance with the National Institutes of Health Guidelines for the Care and Use of Laboratory Animals and were approved by the University of Illinois Institutional Animal Care and Use Committee.

### Drugs

MA [(+)-Methamphetamine hydrochloride (Sigma, Cat # M-8750)] was dissolved in sterile saline and injected i.p. at a dose of 5 mg MA·HCl/kg body weight, four times at 2 h intervals. This dosing schedule was based on reports by Thomas and Khun [7,13] that showed the schedule induced microglial activation. *Escherichia coli *LPS (serotype 0127:B8, Sigma) was dissolved in sterile saline and injected i.p. at 100 μg/kg body weight, a dose that reliably causes fever in mice. Both MA and LPS were diluted such that each dose was equivalent to an injection volume of 0.01 mg/g. Control mice received an equivalent volume of saline.

### Experimental design

#### MA and LPS

To investigate if MA administration affects the inflammatory response to peripheral immune activation, mice received four i.p. injections of either 5 mg/kg MA or saline at 2 h intervals. Twenty-four hours after the first MA injection, mice received i.p. injections of either LPS (100 μg/kg) or saline and Tb and locomotor activity were monitored for the next 24 h. Mice were then killed by CO_2 _asphyxiation and blood and brains were collected for cytokine measurement. To measure cytokines at a time point when mice were actively sick, a separate group of mice not implanted with E-mitters was subjected to the same experimental protocol and killed at 4 h post-LPS.

### Cytokine mRNA measurement by quantitative real-time PCR

Total hypothalamic RNA was isolated using the Arcturus PicoPure™RNA isolation kit as described by the manufacturer. DNase treatment was performed on a PicoPure column with a Qiagen RNase-free DNase set (Qiagen, Valenica, CA). RNA from all other regions was isolated using the Tri Reagent protocol (Sigma, St. Louis, MO). A QuantiTect Reverse Transcription Kit (Qiagen, Valencia, CA) was used for cDNA synthesis with integrated removal of genomic DNA contamination according to the manufacturer's protocol and previously described [26]. Quantitative real-time PCR was performed using the Applied Biosystems (Foster, CA) Assay-on Demand Gene Expression protocol as previously described [26]. In brief, cDNA was amplified by PCR where a target cDNA (IL-6, Mm00446190_m1; IL-1β, Mm00434228_m1; TNFα, Mm00443258_m1; and CD68, Mn00839636_gl) and a reference cDNA (glucose-3 phosphate dehydrogenase, Mm99999915_g1) were amplified simultaneously using an oligonucleotide probe with a 5' fluorescent reporter dye (6-FAM) and a 3' quencher dye (NFQ). PCR reactions were performed in triplicate under the following conditions: 50°C for 2 min, 95°C for 10 min, followed by 40 cycles of 95°C for 15 sec, and 60°C for 1 min. Fluorescence was determined on an ABI PRISM 7900HT-sequence detection system (Perkin Elmer, Forest City, CA). Data were analyzed using the comparative threshold cycle (Ct) method, and results are expressed as fold difference.

### Plasma cytokines

Plasma samples were assayed for IL-1β, TNFα, IL-6, and IL-10 using a multiplex bead-based immunoassay kit combined with a Cytokine Reagent kit as described by the manufacturer (Bio-Rad, Hercules, CA). The multiplex assay was sensitive to <3 pg/ml for IL-1β, TNFα, IL-10, and IL-6. The inter-assay and intra-assay coefficients of variation were <8%.

### Immunohistochemical staining and quantification

A separate group of mice was exposed to the MA regimen and then given either 100 μg/kg of LPS or saline. Four hours after i.p. injection of LPS, mice were killed by CO_2 _asphyxiation and transcardially perfused with heparinized saline followed by 4% paraformaldehyde, and brains were removed. Brains were blocked in 3 parts (rostral, mid and caudal), postfixed over 2 days in 4% paraformaldehyde, and paraffin embedded. Coronal sections (4 μm) were cut on a microtome and every 10^th ^section was stained for Iba1 (ionized calcium binding adapter molecule 1; Wako Chemicals USA, Richmond, VA) at the level of the striatum (Bregma -0.82 mm) and at the hippocampus (Bregma -2.18 mm). Sections were dewaxed and rehydrated through xylene and alcohols and were incubated in citrate buffer, pH 6, and microwaved for 10 min. Endogenous peroxidase was eliminated by incubating sections in 3% H_2_O_2_/methanol for 15 min. Sections were washed in PBS and blocked with 5% normal goat serum in PBS before overnight incubation at 4°C with the primary antibody (1:1200) in 5% blocking serum. The sections were washed, and then incubated with biotinylated goat anti-rabbit antibody (Vector Laboratories, Burlingame, CA) for 1 h. Staining was visualized using the ABC method and 3,3'-diaminobenzidine and sections were counterstained with hemotoxylin. Isotype-matched IgG was used as negative control. Immunostaining was visualized using an Optronix Microfire camera (model S99808, Goleta, CA) attached to a Zeiss Axio Imager A.1 microscope (Gottingen, Germany). The numbers of Iba1-positive cells were counted by two blind observers and verified using ImageJ http://rsbweb.nih.gov/ij/. The number of positive cells was determined in both hemispheres of a given section using a total of four sections per mouse and expressed as number of cells per 100 μm^2^.

### Data Collection and Analysis

Tb and locomotor activity data were collected every 5 min and averaged over 30 minutes. Change in locomotor activity following injection was calculated as the 30-min average at each time point minus the 30-min average at time 0 (the time of injection). Tb and locomotor activity data from LPS experiments were subjected to repeated-measures ANOVA in which Time was a within-subjects measure and MA (MA or Saline) and LPS (LPS or Saline) were between-subjects measures. Central cytokine mRNA levels and peripheral cytokines were analyzed using two-way ANOVA (MA × LPS). When ANOVAs revealed a significant effect of main factors or main factor interactions, differences in treatment group means were tested using Fisher's least-significant differences. All data are presented as means ± SEM.

## Results

### MA administration

**Figure 1:**
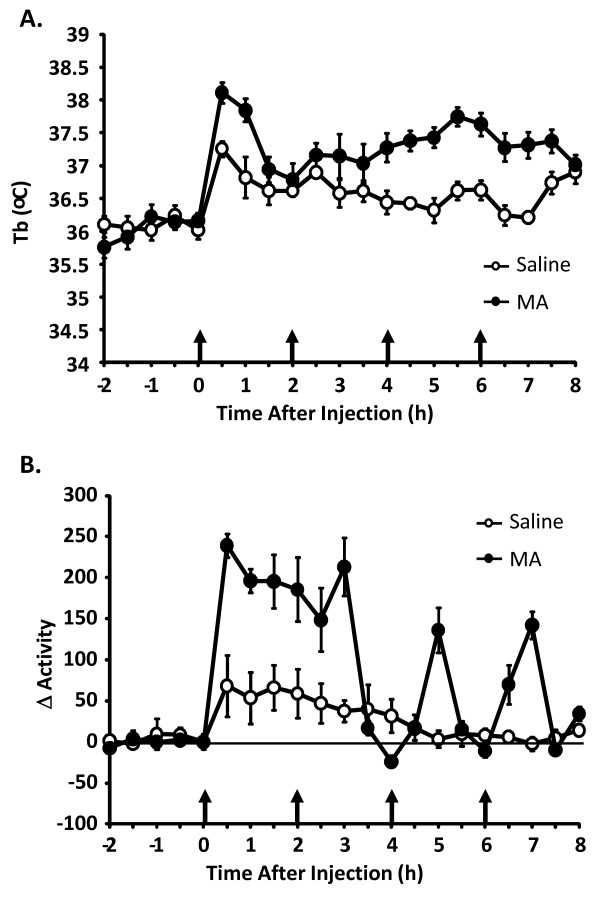
**Body temperature and locomotor activity during methamphetamine administration**. Effect of 4 i.p. injections of MA on Tb (A), and change in locomotor activity (B) Open symbols represent saline controls and closed symbols represent MA administration (n = 16-18 per group). Activity data is presented as 30 minute averages from time of injection. Arrows indicate injection times.

Figure 1A and 1B shows Tb and changes in locomotor activity during MA administration. As expected, there was a main effect of MA for Tb [F (1,30) = 8.022, p < 0.01] and locomotor activity [F (1,30) = 17.27, p < 0.001] whereby MA increased both Tb and locomotor activity compared to saline.

### Methamphetamine administration enhances LPS-induced fever

**Figure 2:**
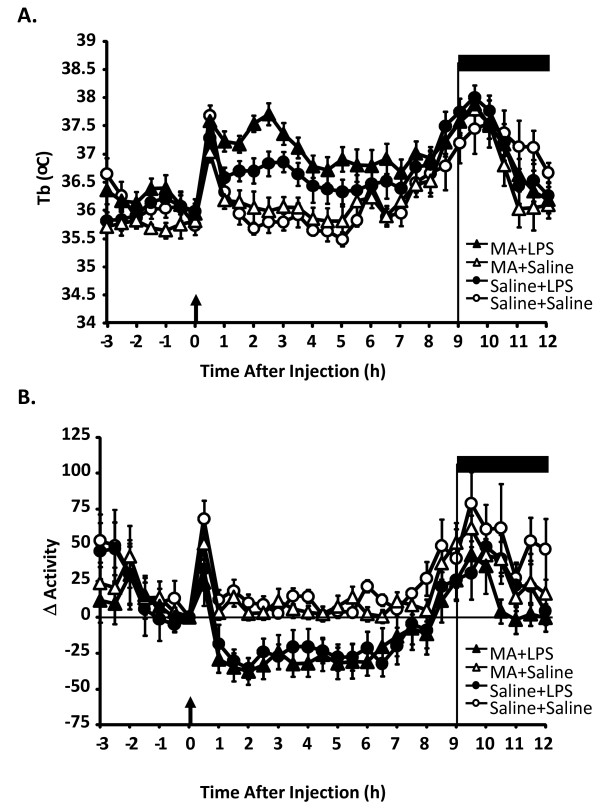
**Body temperature and locomotor response to LPS in mice exposed to methamphetamine**. Tb (A) and locomotor activity (B) after LPS or saline in mice exposed to MA. Mice were exposed to 4 i.p. injections of 0 or 5 mg/kg MA and then injected with 100 μg/kg LPS or saline, 24 h after the first MA injection. Open circles represent saline + saline administration, open triangles represent saline + MA administration, closed circles represent saline + LPS administration and closed triangles represent mice MA + LPS administration (n = 6-10 per group). Arrow indicates time of injection; dark bar indicates lights off.

We next investigated if MA administration would interfere with the febrile response to a peripheral immune stimulus (LPS, 100 μg/kg). Mice received four i.p. injections of MA (5 mg/kg) or saline and then were given i.p. LPS or saline 24 h after the first MA injection. LPS induced an increase in Tb that peaked 3-4 h after injection (Figure 2A). For Tb, there were main effects of LPS [F (1,30) = 25.459, p < 0.001] and MA [F (1,30) = 6.139, p < 0.02]. There was also a trend toward a MA × LPS interaction (p = 0.06) where animals exposed to MA had higher fevers in response to LPS than those given LPS alone. Indeed, the Tb of mice receiving LPS alone peaked at 1.4°C compared to Tb at time 0 while those exposed to MA before LPS administration had a peak fever of 2.2°C.

As expected, there was a main effect of LPS for locomotor activity [F (1,30) = 12.012, p < 0.01] where LPS decreased locomotor activity compared to saline. This LPS-induced decrease in locomotor activity was not influenced by prior exposure to MA (Figure 2B).

### Methamphetamine administration exacerbates the neuroinflammatory response to LPS

**Figure 3:**
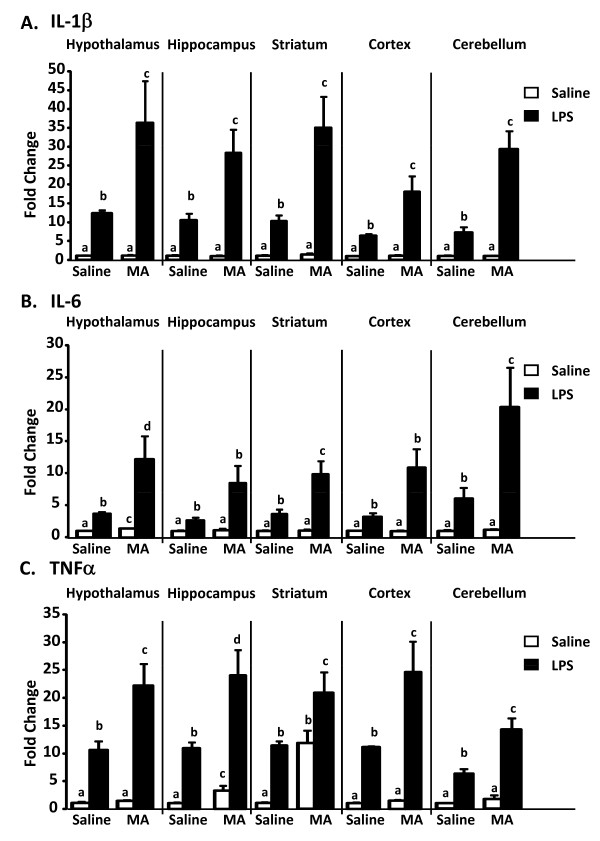
**Cytokine mRNA expression 4 h after LPS in mice exposed to methamphetamine**. IL-1β (A), IL-6 (B), and TNFα (C) mRNA expression in the hypothalamus, hippocampus, striatum, cortex, and cerebellum in mice exposed to MA. Mice exposed to MA were given LPS or MA and brains were collected 4 h post-LPS (n = 4 per group). Means with different letters are at least p < 0.05 different from each other.

To examine the neuroinflammatory response to LPS when mice were actively sick, animals were given four injections of 5 mg/kg MA or saline, followed by LPS or saline 24 h after the first MA injection. Mice were killed at 4 h post-LPS and blood and brains were collected. To examine the global effects of MA and LPS in the brain, five regions were collected: the hypothalamus and hippocampus, which are intimately involved in the sickness response; the striatum and cortex, regions sensitive to MA; and the cerebellum, a region important for motor control. Figure 3A, B and 3C shows changes in inflammatory cytokine mRNA in the five brain regions studied. MA alone increased IL-6 mRNA levels in the hypothalamus (main effect of MA, p < 0.01) and TNFα mRNA in the hippocampus and striatum (main effect of MA, p < 0.01 and p < 0.001, respectively). This trend was also observed in the other regions, although it was not statistically significant. The MA-induced increase in TNFα mRNA was most apparent in the striatum, where MA increased TNFα mRNA levels to the same extent as that of LPS alone (Figure 3C).

LPS increased inflammatory cytokine mRNA in all brain regions examined. For IL-1β mRNA (Figure 3A), there was a MA × LPS interaction whereby MA exposure exacerbated the LPS-induced increase in the hypothalamus [F (1,12) = 6.545, p < 0.03], hippocampus [F (1,12) = 7.972, p < 0.02], striatum [F (1,12) = 8.385, p < 0.02)], cortex [F (1,12) = 8.242, p < 0.02)], and cerebellum [F (1,12) = 19.873, p < 0.001]. A similar interaction was seen for IL-6 mRNA (Figure 3B) in the hypothalamus [F (1,12) = 5.232, p < 0.05], striatum [F (1,12) = 8.169, p < 0.02], cortex [F (1,12) = 6.946, p < 0.03], and cerebellum [F (1,12) = 4.993, p < 0.05]. The MA × LPS interaction was also apparent for TNFα mRNA (Figure 3C) in the hypothalamus [F (1,12) = 6.942, p < 0.03], hippocampus [F (1,12) = 5.14, p < 0.05], cortex [F (1,12) = 5.673, p < 0.04] and cerebellum [F (1,12) = 9.964, p < 0.01].

### Methamphetamine administration increases microglial expression of Iba1

**Figure 4:**
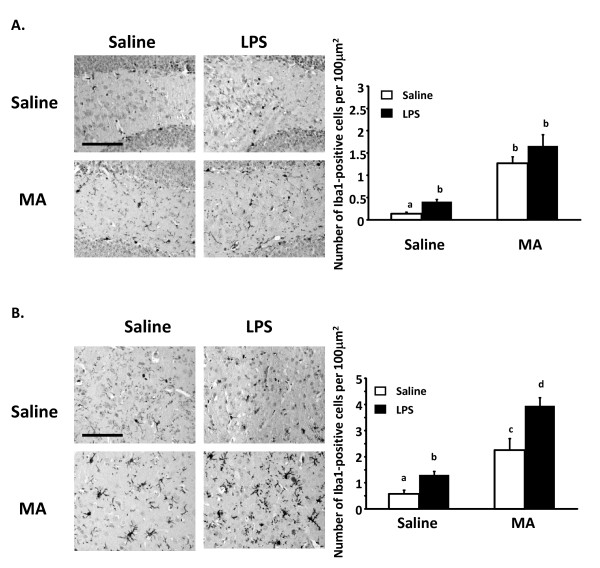
**Iba1 expression 4 h after LPS in mice exposed to methamphetamine**. Immunohistochemical staining of Iba1 in the dentate gyrus of the hippocampus (A) and the striatum (B) of mice exposed to MA prior to LPS administration at 40× magnification. Mice were exposed to four i.p. injections of 0 or 5 mg/kg MA at 2 h intervals and then injected with 100 μg/kg LPS or saline 24 h after the first MA injection. Brains were collected 4 h after LPS administration. Scale bar = 100 μm. Bar graphs express number of Iba1-positive cells per 100 μm^2^.

Based on the results of Thomas and Kuhn (2005), the MA dosing regimen used here was expected to activate microglia. We therefore investigated if MA administration altered the microglial response to LPS. Iba1 is specifically expressed in microglia and expression is particularly enhanced under pathological conditions [27-30]. MA exposure increased Iba1 expression in both the striatum and the hippocampus (main effect of MA, p < 0.001). This MA-related increase was also observed for CD68 mRNA in the hypothalamus, hippocampus, striatum, and cerebellum (data not shown). Furthermore, while there were main effects for MA and LPS in both the hippocampus and straitum (Figure 4A and 4B), there was a MA × LPS interaction in the striatum [F (1,12) = 5.386, p < 0.05] whereby Iba1 expression was further increased when MA exposure was paired with LPS administration.

### Methamphetamine influences plasma cytokines

To investigate the peripheral cytokine response to LPS in MA-exposed mice, blood was collected 4 h post-LPS and plasma cytokines were measured (Table 1). LPS increased plasma IL-10 levels regardless of MA exposure (main effect of LPS, p < 0.01). For IL-1β there was a significant MA × LPS interaction whereby MA alone increased IL-1β while MA plus LPS effectively abolished the LPS-induced increase [F (1,12) = 10.145, p < 0.01]. A similar MA-related inhibition of the LPS-induced increase was seen for TNFα but was not statistically significant (p = 0.08). The opposite trend was observed for plasma IL-6. LPS increased IL-6 in both MA-exposed and non-exposed mice (main effect of LPS, p < 0.01), but there was a trend towards a MA × LPS interaction (p = 0.08) where mice exposed to MA had increased plasma levels of IL-6 than did those given LPS alone. These data suggest that exposure to MA can alter the peripheral inflammatory cytokine response to LPS.

**Table 1:** Plasma cytokines 4 h after LPS in mice exposed to MA.

	0 mg/kg MA		5 mg/kg MA		p		
	
	Saline	LPS	Saline	LPS	MA	LPS	MA × LPS
IL-1β	124.63 ± 47.25	253.4 ± 47.48	226.77 ± 10.97	135.57 ± 12.7	0.82	0.59	0.0078^#^

IL-6	36.61 ± 11.41	662.74 ± 426.8	33.54 ± 9.27	1557.7 ± 196.1	0.08	0.006*	0.08

TNFα	278.61 ± 78.1	501.02 ± 106.5	268.05 ± 33.3	225.49 ± 32.7	0.06	0.22	0.08

IL-10	107.89 ± 28.2	220.43 ± 33.9	111.0 ± 10.57	287.12 ± 69.4	0.42	0.005*	0.46

Mice were given four injections of saline or MA (5 mg/kg) i.p. at 2 h intervals. Twenty-four hours after the first MA injection, mice were given either saline or LPS (100 μg/kg) and blood was collected at 4 h post-injection. *p < 0.01, different from saline controls. ^#^p < 0.01 MA × LPS interaction

## Discussion

MA is a popular drug of abuse that has several central effects and prolonged, or heavy usage can activate the primary inflammatory cells of the brain; microglia. Because MA has been shown to heighten the inflammatory environment of the brain, we hypothesized that MA administration would exacerbate LPS-induced sickness behavior as well as the neuroinflammatory cytokine response. Using a MA regimen expected to activate microglia, we demonstrate that MA administration enhanced LPS-induced fever, and this response was paralleled by a dramatic increase in central cytokine mRNA expression 4 h post-LPS. Moreover, the MA-induced increase in microglial Iba1 expression was even greater in mice exposed to both MA and LPS, suggesting that MA can alter the response of microglia to a subsequent peripheral immune stimulus.

MA-induced microglial activation has been demonstrated in animals given high doses of MA [11-13] and is associated with the production of inflammatory cytokines in the brains of rodents [14-17]. However, little is known concerning the neuroinflammatory consequences of such MA use on a subsequent peripheral immune stimulus. We therefore utilized a MA regimen demonstrated to activate microglia and measured Iba1 expression 4 h after LPS. Microglial Iba1 expression was significantly increased in animals exposed to MA. While MA appeared to increase expression of microglial markers globally, the effect was most apparent in the striatum. Given that MA can greatly affect dopamine nerve endings in the striatum, the robust MA-related staining observed in this region is unsurprising. More interestingly, when LPS was given to mice exposed to MA, Iba1 expression was further increased in this region. This MA-related increase in Iba1 expression was paralleled by increased expression in inflammatory cytokines in brain 4 h after LPS. This suggests that the current model of MA exposure not only activates microglia, but alters them such that they respond more when the peripheral immune system is stimulated.

MA-related microglial responses appear to depend on the dose and schedule used. For instance, there are several reports demonstrating that chronic low-dose MA can attenuate the neurotoxic effects of [31-35], and the microglial response to [13], a subsequent high-dose MA challenge. Our previous study found that while a single administration of 1 mg/kg activated microglia, mice exposed to chronic low-dose MA showed little microglial activation 72 h after the last treatment and responded minimally when exposed to LPS or an additional MA injection [26]. This microglial response was accompanied by an attenuated central inflammatory cytokine response to LPS. The difference between our previous results and our current results lies in the MA dosing regimen; while our previous study was based on a pharmacological dose, the current study utilized a neurotoxic dosing schedule. This schedule has been shown to damage dopamine nerve terminals, promote glial activation, and increase proinflammatory cytokines [7,36,37]. Given the dramatic difference between the two doses and schedules, the difference in the respective elicited responses is not surprising. While we did observe MA-associated increases in IL-6 and TNFα mRNA, these increases (with the exception of TNFα mRNA in the striatum) were small. This is most likely due to the time point of measurement (4 h post-LPS but 16 h after the first MA administration). Possibly, had cytokines been measured soon after MA-exposure, there would have been a MA-related increase. Taken together, it is possible that MA differentially affects the microglial response to a peripheral immune stimulus, either inhibiting or exacerbating the central immune response depending on the dose and schedule.

Peripheral immune stimulation induces cytokines in both the brain and the periphery. In the present study, there was a trend toward MA-related differences in LPS-induced plasma cytokines, indicating that MA influences not only the central cytokine response, but the peripheral response as well. This was particularly true for IL-1β, in which there was a MA × LPS interaction whereby MA alone increased IL-1β but also inhibited the LPS-induced increase in the cytokine. A similar but not significant effect was observed for plasma TNFα. This suggests that while MA administration heightened the central inflammatory response, it concomitantly suppressed the inflammatory cytokine response in the periphery. This differs from our previous study which showed that, while acute administration of 1 mg/kg MA increased plasma IL-1β 4 h post-injection, neither acute nor repeated low-dose MA administration interfered with the LPS-induced increase in plasma cytokines [26]. As in the case of microglia responses, the difference is most likely due to differences in the dose and dosing schedule used. However, the current results are generally consistent with other reports showing suppressive effects of MA on peripheral immune function [21,22]. IL-1β is an important cytokine involved in fever. Both peripheral and central administration of IL-1β induces fever [38,39] and blocking IL-1β attenuates the fever response to LPS [40,41]. Given the bidirectional nature of communication between periphery and brain and the heightened inflammatory environment of the MA-exposed brain, it is possible that the MA-related decrease in LPS-induced IL-1β plasma is an adaptive mechanism that may serve to downregulate signals from the periphery in response to the heightened neuroinflammatory signals in the CNS. Taken together, our data suggest that MA alters the production of both inflammatory and anti-inflammatory cytokines in the periphery while inducing a heightened inflammatory environment in the brain.

Peripheral immune stimulation activates brain microglia and LPS reliably induces cytokines in both the brain and the periphery, but it is this central cytokine response that is essential for the initiation of sickness behavior, which is an integrated, adaptive, and protective response to illness necessary for recovery and survival. MA use is associated with an increased prevalence of HIV infection, hepatitis B and C, fungal infections, and possibly others [1-4]. Given the popularity of MA and its association with immune dysfunction even years after MA use has ceased, it is important to understand how MA influences the immune response. The potential that MA use can exacerbate the neuroimmune response to an invading pathogen presents a serious problem in the human population, since an optimal inflammatory response is necessary for survival and recovery. Furthermore, because neuroinflammation has been demonstrated to be an important mechanism involved in a number of CNS pathologies, it may be possible for MA abuse to exacerbate or hasten the progression of these diseases.

## Competing interests

The authors declare that they have no competing interests.

## Authors' contributions

JBB designed and performed the experiments, analyzed the data, and wrote the manuscript. NLS designed and performed the experiments and edited the manuscript. RWJ oversaw the experimental design and edited the manuscript. All authors have read and approved the final version of the manuscript.
